# Persistence on subcutaneous tocilizumab as monotherapy or in combination with synthetic disease-modifying anti-rheumatic drugs in rheumatoid arthritis patients in Greece (EMBRACE study): a multicenter, post-marketing, non-interventional, observational trial

**DOI:** 10.1007/s10067-024-06916-5

**Published:** 2024-03-12

**Authors:** Panagiotis Athanassiou, Pelagia Katsimbri, Andreas Bounas, Sοusana Gazi, Theodosios Sarikoudis, Victoria Syrigou, Dimitrios Boumpas, EMBRACE Study Group, Athanasios Georgiadis, Ioannis Kallitsakis, Georgios Kampakis, Anna Kandyli, Anastasios Kotrotsios, Eftychia-Maria Koukli, Georgios Ksirogiannis, Aristeidis Lagoudakis, Clio Mavragani, Dimitra Mavridou, Pavlos Merantzis, Theodoros Natskos, Georgios E. Papadimitriou, Dimitrios Petrou, Dimitrios Psaltis, Eleni Tsiakou, Marina Zakalka, Athanasios Ziogas

**Affiliations:** 1 General Hospital of Thessaloniki “Agios Pavlos”, Thessaloniki, Greece; 2 General Hospital of Athens “KAT”, Athens, Greece; 3 “OLYMPION”, General Clinic of Patra, Patra, Greece; 4 Private Practice, Filikis Eterias 9, Kavala, Greece; 5 Roche Hellas S.A, Maroussi, Greece; 6 General University Hospital of Athens “Attikon”, Athens, Greece

**Keywords:** Biological therapies, Medication persistence, Rheumatoid arthritis, Subcutaneous injection, Tocilizumab

## Abstract

**Introduction:**

Rheumatoid arthritis (RA) is a systemic, inflammatory disease affecting multiple organs and causing physical disability over time.

**Objective:**

The primary objective was to evaluate treatment persistence to subcutaneous tocilizumab (TCZ-SC). Additionally, treatment effects on persistence and their associations with clinical and patient-reported outcomes were assessed.

**Method:**

We performed a multicenter, non-interventional, 52-week observational study on 222 patients with moderate or severe RA. Clinical outcomes were evaluated by using disease activity score for 28 joints (DAS28) and European League Against Rheumatism (EULAR) response, and patients’ perceptions were evaluated by using Health Assessment Questionnaire (HAQ), Visual Analog Scale (VAS) for pain, and patient global assessment (PtGA) of disease activity. Safety was assessed throughout the study.

**Results:**

The mean age of the overall cohort was 62.2 ± 12.3 years, and 83.8% were females. Persistence to TCZ-SC was 89.6% at week 24 and 85.1% at week 52 in the overall cohort with slightly increased persistence in the combination group. At week 52, changes from the baseline were − 2.68 in DAS28, − 0.76 in HAQ, − 43.21 in VAS pain, and − 41.66 in PtGA (*p* < 0.0001 for all). Moderate and good EULAR response was achieved in 83.2% of patients. Non-serious and serious adverse events occurred in 18.5% and 3.2% of the participants, respectively.

**Conclusions:**

The current study confirms the favorable safety and effectiveness of TCZ-SC as well as its acceptability by RA patients in Greece, with sustained high persistence rates up to 52 weeks. TCZ-SC offers a sustainable treatment response in RA.

**Table:** 

**Key Points** • *Based upon clinical and patient-reported outcomes, TCZ-SC is a highly effective and safe treatment modality in patients with moderate-to-severe RA.* • *Persistence to TCZ-SC was high throughout the study, both as monotherapy and in combination with csDMARDs.* • *TCZ-SC is effective both as monotherapy and when used in combination with other csDMARDs regardless of the line of treatment.*

**Supplementary Information:**

The online version contains supplementary material available at 10.1007/s10067-024-06916-5.

## Introduction

Rheumatoid arthritis (RA) is a common progressive systemic disease characterized by inflammation of the synovium leading to irreversible joint destruction and patient disability [1, 2]. Although cardinal signs of RA are related to the musculoskeletal system, extra-articular involvement, such as by the pulmonary and cardiovascular system, accompanies peripheral joint manifestations [3, 4]. The clinical course of RA is most commonly insidious [5]. The chance to develop a severe disability in RA is reported in nearly 33% of afflicted patients in 20 years, with less than 50% of patients being able to continue working 10 years following diagnosis [5].

The treatment of RA is a comprehensive approach including effective inflammation control by pharmacological agents as well as educational programs and psychological support to prevent quality of life deterioration and to improve everyday activities [5, 6]. The goal of RA treatment is to attain sustained remission or low disease activity based on shared decision-making between patients and healthcare professionals [2]. Biological disease-modifying antirheumatic drugs (bDMARDs) targeting cytokines that regulate chronic inflammation have opened a new treatment era, especially in patients who cannot tolerate conventional synthetic DMARDs (csDMARDs) [7, 8]. Monoclonal antibodies against tumor necrosis factor-alpha (TNF-α) have been widely used in clinical practice, but are not effective in all patients [9]. Approximately one-third of patients receiving a TNF inhibitor (anti-TNF) experience primary treatment failure or inefficacy according to the American College of Rheumatology (ACR) criteria 20 (ACR20), while more than 50% fail to achieve at least an ACR50 response [6]. Moreover, efficacy is lost over time or patients develop adverse events [6]. These limitations have prompted an investigation into new targets and the development of therapies with alternative mechanisms of action.

Interleukin-6 (IL-6) is a glycoprotein that is well-recognized for its role in the acute-phase inflammatory response, bone metabolism and remodeling, reproductive system, cancer progression, autoimmunity, and metabolic alterations in the liver [10–14]. High IL-6 levels have been detected in the synovial fluid of patients with various forms of inflammatory arthritis including RA [15]. After the identification of the signaling pathways and biological activities of IL-6 and its receptor (IL-6R), researchers decided to target IL-6R through novel therapeutic agents. It has been found that the concentrations of Il-6R have less interpersonal variability, a factor that might potentially allow for the simplification of dosing and selection of the regimen [16]. Employing the advances in biotechnology has been developed [16].

Tocilizumab (TCZ) is a humanized monoclonal antibody of the immunoglobulin G1 subclass targeting IL-6R [16]. TCZ has been shown to improve the clinical signs and symptoms, laboratory results, and radiological findings in refractory RA as monotherapy or in combination with conventional synthetic DMARDs (csDMARDs) [16] [2, 13, 16, 17]. Other than intravenously, TCZ can be administered subcutaneously (SC) once monthly [18, 19]. Despite the high treatment efficacy rates of biological DMARDs (bDMARDs), persistence to treatment is lower than anticipated in real-life settings and registries compared to randomized studies [20]. In the Danish DAINBO registry study of RA patients that initiated bDMARD treatment, nearly 70% of patients were persistent to treatment with bDMARDs, with 19% of patients achieving ACR70 after 6 months of treatment [21]. The availability of dDMARDs that are self-administered via SC injection has empowered patients and are expected to improve the overall patient experience and, thereby, persistence to treatment. However, there are challenges to this mode of administration, such as patient phobia due to self-administration, particularly in elderly patients [22].

**Fig. 1:**
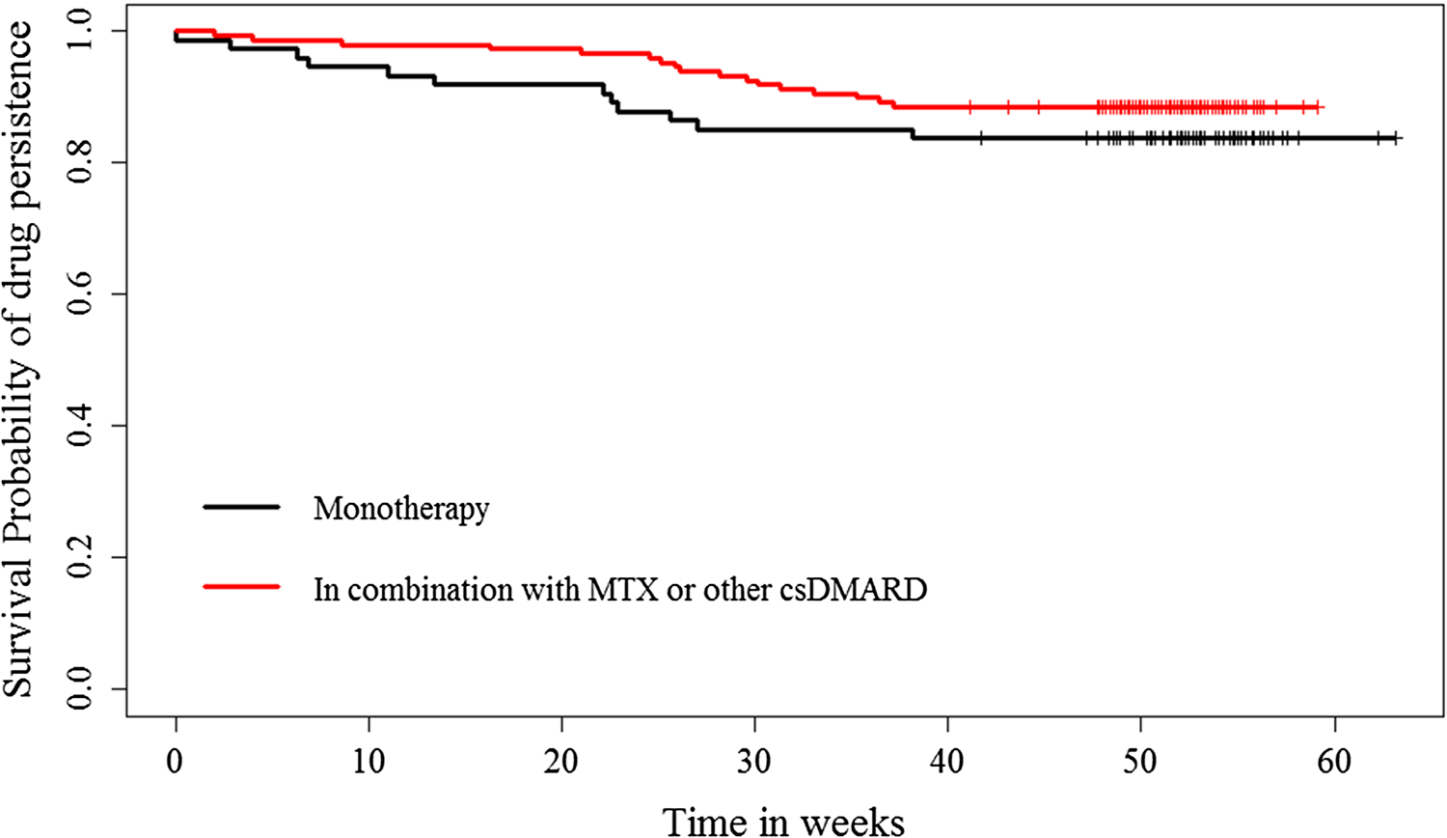
Kaplan-Meier estimates of TCZ SC persistence per treatment group. Censored observations are marked with “+”

Persistence to treatment is critical for all chronic diseases and is used as a surrogate for treatment effectiveness [21, 23]. To date, there are limited data on the persistence to TCZ and particularly TCZ-SC under real-life conditions in Greece. The objective of this study was to evaluate treatment persistence among RA patients under conditions of routine clinical practice and to determine the impact of TCZ-SC on patient-reported outcomes (PROs) as well as clinical efficacy parameters in the real-life setting. We also aimed to identify the impact of baseline patient and/or disease characteristics on persistence to treatment (Fig. 1, Table 5).

## Material and methods

### Setting, study design, and population

The present observational study was conducted in 23 sites across Greece. Overall, 222 adult patients with moderate or severe RA, according to the revised 1987 ACR or 2010 ACR/EULAR classification criteria, who started receiving treatment with TCZ-SC (monotherapy or combination) up to 4 weeks at the physician’s discretion were enrolled after written consent was provided [24, 25]. Patients who had been receiving tocilizumab for more than 4 weeks prior to enrollment or received tocilizumab in past treatments or had received treatment with any investigational agent within 4 weeks (or 5 half-lives of investigational agent, whichever is longer) of starting treatment with TCZ-SC or had a history of autoimmune disease (other than autoimmune disease of the endocrine system) or any joint inflammatory disease other than RA were excluded from this study. Participants were evaluated at baseline, week 24, and week 52. Ethics approval was obtained by the corresponding Hospital Ethics Committee/Institutional Review Board. Informed consent was obtained from all subjects. This study was conducted according to the Declaration of Helsinki and Good Clinical Practice.

### Data collection and outcome variables

Patient and disease characteristics, routine general and inflammatory blood tests, clinical and patient-reported outcomes (disease activity score-28 (DAS-28), EULAR response, Health Assessment Questionnaire (HAQ), Visual Analog Scale (VAS) for pain, and patient global assessment of disease activity (PtGA)), information about attendance in a patient support program (PSP) for TCZ-SC, and information about adverse events were recorded during the study visits.

The primary efficacy endpoint was the percentage of patients still on treatment with TCZ-SC at week 52. Clinical and PROs were analyzed at weeks 24 and 52. Clinical outcomes were evaluated by determining the proportion of patients in remission or with low disease activity (DAS28 < 2.6 or DAS28 ≤ 3.2, respectively) and patients with good/moderate EULAR response criteria [26, 27]. The PRO measures used were the ΗΑQ score (decrease by ≥ 0.3 and ≥ 0.5, converted to the original tool), VAS pain (improvement of at least 10 mm in a 100 mm scale), and PtGA (improvement of at least 15 mm in a 100 mm VAS scale) [28–31].

Associations between treatment persistence and clinical outcome parameters, PROs, involvement in a PSP, and patient and disease characteristics were assessed. The persistence in monotherapy and combination groups was also investigated.

The main safety parameters were the incidence of adverse events (AEs), serious adverse events (SAEs), non-serious adverse reactions, serious adverse drug reactions (SADRs), injection site reactions (ISRs), and adverse events of special interest (AESIs). The incidence, severity, and type of these safety events were compared between persistent vs. non-persistent patients and between monotherapy vs. combination therapy groups.

### Statistical analysis

#### Statistical analysis was performed via SAS

Descriptive statistics and standard deviation (SD) were used to compare demographic and disease characteristics, laboratory values, medical history, and treatment responses. Exploratory statistical testing and modeling were performed by using paired *t*-tests and ANOVA. All tests were two-sided and carried out with a 5% α-error rate.

Continuous efficacy parameters and PROs, their changes from baseline, and concomitant glucocorticoids (GCs) and csDMARDs were summarized at each time point by mean, SD, and median. Categorical efficacy parameters including the EULAR response rate and the involvement in a TCZ PSP were summarized by numbers and percentages (*n*, %).

The proportion of patients experiencing at least one AE was estimated with 95% confidence intervals (CIs). Adverse event rates per 100 patient-years were calculated separately for patient subpopulations. Logistic univariate regression analysis was performed to assess the impact of baseline characteristics and TCZ-SC persistence at the end of the study. The level of significance was set at 5%.

## Results

The FAS included 222 patients, of whom 199, 192, and 185 patients completed 24 weeks, 40 weeks, and 52 weeks of treatment with TCZ-SC.

Of the study population (*n* = 222), 186 were women (83.8%). The mean (SD) age was 62.2 years (± 12.3) with approximately 60% of patients being ≥ 60 years. The mean duration of RA was 6.9 ± 7.3 years. At baseline, the mean DAS28 score was 5.6 ± 1.1 for the overall study population, and 9% of all patients had good EULAR response. The most common extra-articular manifestation was rheumatoid nodules (34.2%). Before enrollment, 74.3% of subjects had received at least one RA treatment excluding TCZ and 49.1% were bDMARD-naïve. The baseline demographics of patients and disease characteristics are presented in Table 1.

**Table 1:** Baseline characteristics of the study population

Demographics	
Age (years)—mean ± SD	62.2 ± 12.3
Female patients, *n* (%)	186 (83.8)
BMI (kg/cm^2^), mean ± SD	27.5 ± 5.3
Smokers, *n* (%)	48 (21.6)
Alcohol consumption, *n* (%)	36 (16.2)
RA status	
RA duration (years), mean ± SD	6.9 ± 7.3
RF seropositive, *n* (%)	127 (57.2)
Anti-CCP seropositive—no (%)	87 (41)
DAS28 score, mean ± SD	5.6 ± 1.1
RA-related treatment	
Biologic naïve, *n* (%)	109 (49.1)
No. of prior biologic treatments, median	1.00
Concomitant MTX or other csDMARDs use, *n* (%)	148 (66.7)
MTX dose (mg/week), mean ± SD	18.7 ± 12.1
Concomitant oral steroids, *n* (%)	133 (59.9)
Daily glucocorticoid dose, mean ± SD	6.5 ± 3.8

### Efficacy outcomes

During the study, 66.7% of patients received combination treatment with methotrexate (MTX) or other csDMARDs, and 33.3% received monotherapy with TCZ-SC.

Patients persistent to treatment with TCZ-SC comprised 89.6% (95% CI; 79.9–98.2%) of the population at week 24 and 85.1% (95% CI; 74.3–95.9%) at week 52. Overall, 6.3% of patients persistent to treatment were enrolled in the PSP. Persistence to TCZ-SC was analyzed in subgroups and was found to be statistically higher in patients receiving combination treatment at both weeks 24 and 52, though no difference was observed per line of treatment (Table 2; Fig. 1 in Supplementary Material). Disease recurrence was observed in 5% and 11.5% of patients under TCZ-SC at the same study time points (Fig. 2 in Supplementary Material).

**Table 2:** Drug persistence according to monotherapy vs. combination therapy and line of treatment at study timepoints

	Drug persistence^*^—*n* (%)	
	Week 24	Week 52
Treatment—no (%)	***N* = 199**	***N* = 189**
Monotherapy (*n* = 74)	63/74 (85.1)	58/74 (78.4)
Combination therapy (*n* = 148)	136/148 (91.9)	131/148 (88.5)
Log rank test of equality of survival distributions between treatment groups	*p* = 0.022	*p* = 0.017
Line of treatment, *n* (%)	***N* = 199**	***N* = 189**
TCZ SC 1st line (naïve) (*n* = 109)	98/109 (89.9)	95/109 (87.2)
TCZ SC 2nd line (*n* = 65)	58/65 (89.2)	54/65 (83.1)
Other lines of treatment (*n* = 48)	43/48 (89.6)	40/48 (83.3)
Log rank test of equality of survival distributions between line of treatment groups	0.372^†^	0.283^†^

^*^Persistence is defined as the number and proportion of patients still on treatment with TCZ SC at a specific timepoint. ^†^Pooled over strata (overall *p* value)

At baseline, high disease activity (DAS > 5.1) was reported for 72.5% of the overall cohort and was decreased to 5.9% and 12% at weeks 24 and 52, respectively. Compared to baseline, DAS28 values were statistically significantly improved in both monotherapy and combination therapy groups (Table 3).

**Table 3:** Clinical outcome assessments for overall, monotherapy, and combination therapy groups during the study

	Baseline visit (week 0) *N* = 222	Observational period (week 24) *N* = 216	Study completion (week 52) *N* = 208
Overall study population			
DAS28 score	***N* = 222**	***N* = 199**	***N* = 208**
Mean ± SD	5.6 ± 1.1	3.2 ± 1.2	2.9 ± 1.6
Median [IQR]	5.7 [5.0–6.3]	3.0 [2.4–4.0]	2.5 [1.7–3.8]
Change in DAS28 score from baseline (mean, 95% CI, *p* value)		− 2.4, (− 2.6, − 2.2), ***p* < 0.0001**	− 2.7, (− 2.9, − 2.5), ***p* < 0.0001**
DAS28 score/RA activity, *n* (%)			
DAS28 < 2.6 (remission)	7 (3.2)	74 (37.2)	110 (52.9)
2.6 ≤ DAS28 ≤ 3.2 (low disease activity)	1 (0.5)	38 (19.1)	23 (11.1)
3.2 ≤ DAS28 ≤ 5.1 (moderate activity)	53 (23.9)	74 (37.2)	50 (24)
DAS28 > 5.1 (high activity)	161 (72.5)	13 (5.9)	25 (12)
EULAR response, *n* (%)	***N* = 222**	***N* = 199**	***N* = 208**
Good	9 (4.1)	109 (54.8)	116 (55.8)
Moderate	87 (39.2)	78 (39.2)	57 (27.4)
No response	126 (56.8)	12 (6)	35 (16.8)
Monotherapy group			
DAS28 score	***N* = 74**	***N* = 63**	***N* = 64**
Mean ± SD	5.7 ± 1.1	3.0 ± 1.2	2.4 ± 1.6
Median [IQR]	5.8 [5.2–6.4]	2.7 [2.3–3.7]	2.1 [1.1–3.4]
Change in DAS28 score from baseline (mean, 95% CI, *p* value)	-	− 2. 8, (− 3.1, − 2.4), ***p* < 0.0001**	− 3.4, (− 3.8, − 2. 9), ***p* < 0.0001**
DAS28 score/RA activity, *n* (%)			
DAS28 < 2.6 (remission)	4 (5.4)	29 (46)	42 (65.6)
2.6 ≤ DAS28 ≤ 3.2 (low disease activity)	0 (0)	11 (17.5)	6 (9.4)
3.2 ≤ DAS28 ≤ 5.1 (moderate activity)	12 (16.2)	19 (30.2)	10 (15.6)
DAS28 > 5.1 (high activity)	58 (78.4)	4 (6.3)	6 (9.4)
EULAR response, *n* (%)	***N* = 74**	***N* = 63**	***N* = 64**
Good	6 (8.1)	43 (68.3)	45 (70.3)
Moderate	33 (44.6)	18 (28.6)	13 (20.3)
No response	35 (47.3)	2 (3.2)	6 (9.4)
Combination therapy group			
DAS28 score	***N* = 148**	***N* = 136**	***N* = 144**
Mean ± SD	5.5 ± 1.1	3.3 ± 1.1	3.1 ± 1.6
Median [IQR]	5.7 [4.9–6.2]	3.1 [2.4–4.0]	2.8 [1.9–4.1]
Change in DAS28 score from baseline (mean, 95% CI, *p* value)	-	− 2.3, (− 2.5, − 2.0), ***p* < 0.0001**	− 2.4, (− 2.6, − 2.1), ***p* < 0.0001**
DAS28 score/RA activity—no (%)			
DAS28 < 2.6 (remission)	3 (2)	45 (33.1)	68 (47.2)
2.6 ≤ DAS28 ≤ 3.2 (low disease activity)	1 (0.7)	27 (19.9)	17 (11.8)
3.2 ≤ DAS28 ≤ 5.1 (moderate activity)	41 (27.7)	55 (40.4)	40 (27.8)
DAS28 > 5.1 (high activity)	103 (69.6)	9 (6.6)	19 (13.2)
EULAR response, *n* (%)	***N* = 148**	***N* = 136**	***N* = 144**
Good	3 (2)	66 (48.5)	71 (49.3)
Moderate	54 (36.5)	60 (44.1)	44 (30.6)
No response	91 (61.5)	10 (7.4)	29 (20.1)

In the overall study population, the DAS28 score decreased from 5.6 to 3.2 at week 24 and to 2.9 at week 52 with remission rates of 37.2% and 52.9%, respectively (Table 3). Over half of the study population attained a good response according to the EULAR criteria at weeks 24 and 52 (Table 3). Statistically significant improvements in DAS28 values and good EULAR response were detected in both monotherapy and combination groups (Table 3) and in patients who received TCZ-SCas first line (TCZ-SC naïve), as second and other lines of treatment (Supplementary Table 2).

The baseline HAQ score recorded by participants described an initial mild to severe disability. A statistically significant improvement in the HAQ score was recorded at week 24 which continued to improve throughout week 52. HAQ ≥ 0.5 improvement was observed in 64.4% and 66.5% of patients at weeks 24 and 52, respectively (Table 4). The same significant improvement using the HAQ score was also observed both in monotherapy and combination therapy groups and in all treatment lines. In accordance with this, the patient’s assessment of their symptoms, using the PtGA and VAS tools, showed a statistically significant improvement in both scores at weeks 24 and 52. At week 52, 90.5% of patients reported a VAS score ≥ 10 mm, and 87.9% had a PtGA score ≥ 15 mm (Table 4). Statistically significant improvements were sustained in HAQ ≥ 0.5, VAS pain score ≥ 10 mm, and PtGA ≥ 15 mm independent from monotherapy or combination therapy or line of treatment.

**Table 4:** Change from the baseline in HAQ, VAS pain, and PtGA scores for the overall study population at study timepoints

	Baseline visit (week 0)	Observational period (week 24)	Study completion (week 52)
HAQ score	***N* = 222**	***N* = 199**	***N* = 208**
Mean ± SD	1.3 ± 0.6	0.7 ± 0. 6	0.6 ± 0.6
Median [IQR]	1.4 [0.9–1.8]	0.5 [0.3–0.9]	0.4 [0.1–0.9]
Change in HAQ score from baseline (mean, 95% CI, *p* value)		− 0.7, (− 0.8, − 0.6), ***p* < 0.0001**	− 0.8, (− 0.9, − 0.7), ***p* < 0.0001**
Improvement in patients’ health (expressed in decrease in HAQ score from baseline), *n* (%)			
HAQ < 0.3		59 (29.6)	59 (28.6)
0.3 ≤ HAQ < 0.5		12 (6)	10 (4.9)
HAQ ≥ 0.5		128 (64.4)	137 (66.5)
VAS for pain score, mm	***N* = 222**	***N* = 199**	***N* = 208**
Mean ± SD	68.2 ± 16.4	30.9 ± 21.8	24.8 ± 24.3
Median [IQR]	70.0 [60.0–80.0]	23.0 [13.0–45.0]	16.0 [6.0–39.5]
Change in VAS pain scale from baseline (mean, 95% CI, *p* value)		− 37.5, (− 40.8, − 34.1), ***p* < 0.0001**	− 43.2, (− 46.8, − 39.6), ***p* < 0.0001**
Improvement of patients’ pain from baseline (expressed in decrease in VAS scale), *n* (%)			
VAS score < 10 mm		17 (8.8)	19 (9.5)
VAS score ≥ 10 mm		176 (91.2)	182 (90.5)
PtGA score, mm	***N* = 222**	***N* = 199**	***N* = 208**
Mean ± SD	66.5 ± 15.9	30.4 ± 21.1	24.7 ± 24.1
Median [IQR]	70.0 [60.0–80.0]	22.0 [14.0–45.0]	18.0 [6.0–39.5]
Change in PtGA scale from baseline (mean, 95% CI, *p* value)		− 35.7, (− 39.0, − 32.5), ***p* < 0.0001**	− 41.7, (− 45.4, − 38.0), ***p* < 0.0001**
Improvement of patients’ QoL from baseline (expressed in decrease in PtGA score), *n* (%)			
PtGA score < 15 mm		27 (14.3)	24 (12.1)
PtGA score ≥ 15 mm		162 (85.7)	174 (87.9)

Significant associations were determined between drug persistence and clinical outcome parameters (DAS28 score and EULAR response) both at week 24 and week 52. However, statistically significant associations between drug persistence, HAQ, VAS pain, and PtGA scores were determined only at week 52 (*p* < 0.001) (Table 5).

**Table 5:** Associations between drug persistence, and clinical and patient reported outcome measures (PROs) at the study timepoints

DAS28 score attainment, *n* (%)	Drug persistence at 52 weeks, *n* (%)	
	Observational period—week 24	Study completion—week 52
DAS28 < 2.6 (remission)	73 (67)	108 (82.4)
2.6 ≤ DAS28 ≤ 3.2 (low disease activity)	36 (33)	23 (17.6)
*X*^2^ value^*^	8.991	54.948
*p* value^*^	0.029	** < 0.0001**
Phi^**^	0.215	0.514
EULAR response rate attainment, *n* (%)		
No response	11 (5.9)	26 (13.7)
Moderate response	70 (37.4)	50 (26.5)
Good response	106 (56.7)	113 (59.8)
*X*^2^ value^*^	6.053	18.264
*p* value^*^	0.048	** < 0.0001**
Phi^**^	0.176	0.296
Drug persistence at 52 weeks, *n* (%)^‡^		
Decrease in HAQ score, *n* (%)	Observational period—week 24	Study completion—week 52
HAQ < 0.3	39 (22.4)	30 (17.4)
0.3 ≤ HAQ < 0.5	12 (6.9)	8 (4.7)
HAQ ≥ 0.5	123 (70.7)	134 (77.9)
*X*^2^ value^*^	4.648	16.525
*p* value^*^	0.098	** < 0.0001**
Cramer’s V^**^	0.160	0.300
Decrease in VAS score, *n* (%)		
VAS score < 10 mm	14 (7.7)	11 (6)
VAS score ≥ 10 mm	167 (92.3)	172 (94)
*X*^2^ value^*^	2.947	28.282
*p* value^*^	0.139	** < 0.0001**
Phi^**^	0.125	0.375
Decrease in PtGA score, *n* (%)		
PtGA score < 15 mm	24 (13.5)	15 (8.3)
PtGA score ≥ 15 mm	154 (86.5)	165 (91.7)
*X*^2^ value^*^	3.559	26.670
*p* value^*^	0.093	** < 0.0001**
Phi^**^	0.138	0.367

^*^Pearson’s Chi-square test is performed. If one or more of the cells has an expected frequency of five or less, Fisher’s exact test is used (Fisher’s exact test does not have a “test statistic”). ^**^Measures the strength of association between two variables. ^‡^Patients with changes in HAQ score from baseline (W24-W0, W52-W0)

The between-group comparison and regression analysis of multiple variables did not show an association with TCZ-SC persistence with the type and line of treatment or the participation in a PSP but discovered a significant result regarding the presence of extra-articular manifestations. Glucocorticoids were administered to 60.4% of the baseline population, decreasing to 55.9% at week 52, with 14.9% being able to reduce or discontinue their glucocorticoid dose at week 24.

### Safety

Overall, 18.5% of study participants (*n* = 41) experienced AE(s), 3.2% (*n* = 7) SAE(s), while 6.3% (*n* = 14) had ADR(s) (Supplementary Material Table 3). When comparing monotherapy vs. combination-therapy groups, a statistically significant higher incidence of injection site reactions (ISR) and deaths was observed in the monotherapy group compared to the combination therapy group (12.1% vs. 0.7%, respectively, for ISR, *p* = 0.013; 6.1% vs. 0%, respectively, for deaths, *p* = 0.033) (Supplementary Material Table 4). Regardless of the incidence of AEs, in the majority of participants, TCZ treatment did not affect total cholesterol or LDL-C levels (Supplementary Material Table 4). In the full population, the most common AEs encountered were dyslipidemia (2.3%), rash (1.8%), and leukopenia (1.4%) (Supplementary Material Table 5). Regarding laboratory evaluations, clinically significant abnormal results were only noted in a few study participants throughout the study. The most common clinically significant laboratory abnormalities at both week 24 and week 52 involved liver enzymes and lipid levels (reported in ≤ 1% of participants (Supplementary Material Table 6).

Per treatment group (monotherapy or combination treatment), adverse events of special interest (AESI) or SAEs were rarely reported (≤ 4.1% of participants per treatment group) (Supplementary Material Table 7 and Table 8). Regarding persistence (persistent vs. non-persistent participants), the incidence of AEs, treatment-related AEs, SAEs, ISRs, and deaths were statistically significantly higher in the non-persistent group of participants compared to the persistent group (Supplementary Material Table 9 and 10).

The annualized rate for AEs, SAEs, and AESI per 100 patient-years was low at 1.51, 1.43, and 2.13, respectively (Supplementary Material Table 11).

AEs were the main reason for TCZ discontinuation (permanent or temporary) in 8.1% of the study participants (Supplementary Material Table 12). The most frequently reported AEs leading to TCZ-SC discontinuation were rash, leukopenia, and diarrhea. Only permanent tocilizumab dose discontinuations were noted in the non-persistent and the monotherapy treatment groups (Supplementary Material Table 4 and 10).

## Discussion

The present study contributed insights on the real-life use, safety, and efficacy of TCZ-SC as well as patient-perceived effect of treatment on quality of life from a cohort of patients who were enrolled in Greece and initiated treatment with TCZ-SC within 4 weeks from enrollment. The majority of patients were persistent to TCZ-SC throughout the study duration. The overall persistence to TCZ-SC was 89.6% at week 24 and 85.1% at week 52, with a recurrence-free rate of 88.5%. These rates are comparable to the literature-reported data for persistence to bDMARDs under real-life conditions [19, 23, 32, 33]. The high rates of persistence were sustained throughout the study up to week 52 across treatment groups, namely, for the monotherapy group 78.4%, combination therapy group 88.5%, and in biologic naïve patients 87.2%. Higher persistence rates for a systematic review of 43 observational studies, conducted both in EU and US, that investigated the adherence and persistence to biologics in chronic inflammatory diseases, such as RA, psoriasis, and psoriatic arthritis (PsA), reported that the range of continuation (persistence) to treatment at 12 months ranged from 32.0 to 90.9%, with higher treatment continuation rates when co-administration with methotrexate or other DMARDs was reported [33].

For many chronic immune system diseases, the availability of IV and SC administration modes aims to ensure that patients are provided with options that are acceptable in order to attain long-term persistence to treatment [34]. Patient preference to treatment administration mode is subjective and can be influenced by factors such as age and, among older individuals, ease of administration (dexterity) [35, 36]. No issues with ease of treatment administration were reported. Based on the review of relevant literature references, factors that contribute towards preference to SC administration include the convenience and comfort of at-home treatment, lack of a requirement to prepare the medicine, the reduced chance of medication error, and ready-to-use syringes with the correct dose, whereas IV infusion preference is mainly driven by fear for self-injection, the feeling of safety due to hospital administration, and opportunity for interactions with the medical team [34, 37–40].

Persistence to treatment is a critical component of effectiveness. The clinical outcome measurements indicated sustained statistically significant improvement of disease severity across a range of disease-related parameters in addition to acceptable tolerability to TCZ-SC in patients with moderate-to-severe RA. These data are in agreement with real-world published evidence [18, 41–45]. In the present study, the majority of patients achieved remission according to the DAS28 score and a good EULAR response. During the study, no unexpected adverse events and no safety concerns were identified relevant to the available safety information on TCZ [18, 41–45]. The incidence of adverse events related to lipid levels, hematology, and infections was low, which is in agreement with published data [43, 46, 47]. The percentage of TCZ-SC discontinuation due to safety reasons was comparable to published evidence from real-life studies [43]. There were significant associations between the persistence and clinical outcome parameters both at weeks 24 and 52, as well as PROs at the end of the study.

This study had some inherent limitations that pertain to its design and concern the lack of control group and formal sample size calculation. The effect of missing data in the analysis of retrospective observational studies cannot be ruled out. Compliance to SC treatment, which could interfere with the study assessments, as opposed to supervised intravenous infusions was not determined. In addition to the aforementioned factors, the conduct of the study in a single country poses limitations on the external validity of its results. Treatment decisions were at the discretion of the treating physicians. However, the findings of this study contribute valuable information regarding real-life patterns of use of TCZ-SC in Greece. The investigators’ decision-making to administer TCZ-SC was based on current medical practice and preceded the consideration of the patient’s eligibility for enrollment into the study.

## Conclusion

The present study contributes national data from Greece on the real-life safety and effectiveness of TCZ-SC when used either as monotherapy or combination therapy in patients with moderate to severe RA, as well as on patient persistence to treatment under conditions of routine clinical practice. In agreement with published data, TCZ-SC is an effective, safe, and acceptable treatment option for RA patients. Patient acceptability to treatment is a critical component for long-term disease management in patients with chronic immune conditions. The findings of this study could prompt further investigation to determine the optimal TCZ treatment administration on eligible RA patients in Greece under real-life conditions with the aim of delivering personalized treatment options that can maximize persistence to treatment.

### Supplementary Information

Below is the link to the electronic supplementary material.

Supplementary file1 (DOCX 210 KB)

## Data Availability

Additional data is available upon legitimate request.

## References

[1] Smolen JS, Aletaha D, McInnes IB (2016). Rheumatoid arthritis. Lancet.

[2] Smolen JS, Landewé RBM, Bergstra SA (2023). EULAR recommendations for the management of rheumatoid arthritis with synthetic and biological disease-modifying antirheumatic drugs: 2022 update. Ann Rheum Dis.

[3] Crowson CS, Liao KP, Davis JM (2013). Rheumatoid arthritis and cardiovascular disease. Am Heart J.

[4] Megan S, Bridget FC, Lawrence AH (2015). Rheumatoid arthritis-associated lung disease. Eur Respir Rev.

[5] Brooks P (1993). Clinical management of rheumatoid arthritis. The Lancet.

[6] Dieppe P (2002). Epidemiology of the rheumatic diseases second edition. AJ Silman, MC Hochberg (eds). Oxford: Oxford University Press, 2001, pp. 377, £95.00. ISBN: 0192631497. Int J Epidemiol.

[7] Bathon JM, Martin RW, Fleischmann RM (2000). A comparison of etanercept and methotrexate in patients with early rheumatoid arthritis. N Engl J Med.

[8] Maini RN, Breedveld FC, Kalden JR (1998). Therapeutic efficacy of multiple intravenous infusions of anti-tumor necrosis factor alpha monoclonal antibody combined with low-dose weekly methotrexate in rheumatoid arthritis. Arthritis Rheum.

[9] Rubbert-Roth A, Finckh A (2009). Treatment options in patients with rheumatoid arthritis failing initial TNF inhibitor therapy: a critical review. Arthritis Res Ther.

[10] Keller ET, Wanagat J, Ershler WB (1996). Molecular and cellular biology of interleukin-6 and its receptor. Front Biosci.

[11] Hirano T (1992). The biology of interleukin-6. Chem Immunol.

[12] Metzger S, Hassin T, Barash V (2001). Reduced body fat and increased hepatic lipid synthesis in mice bearing interleukin-6-secreting tumor. Am J Physiol Endocrinol Metab.

[13] Tamura T, Udagawa N, Takahashi N (1993). Soluble interleukin-6 receptor triggers osteoclast formation by interleukin 6. Proc Natl Acad Sci U S A.

[14] Taub R (2003). Hepatoprotection via the IL-6/Stat3 pathway. J Clin Invest.

[15] Houssiau FA, Devogelaer JP, Van Damme J (1988). Interleukin-6 in synovial fluid and serum of patients with rheumatoid arthritis and other inflammatory arthritides. Arthritis Rheum.

[16] Choy EH, De Benedetti F, Takeuchi T (2020). Translating IL-6 biology into effective treatments. Nat Rev Rheumatol.

[17] Smolen JS, Landewé RBM, Bijlsma JWJ (2020). EULAR recommendations for the management of rheumatoid arthritis with synthetic and biological disease-modifying antirheumatic drugs: 2019 update. Ann Rheum Dis.

[18] Ogata A, Kato Y, Higa S (2019). Subcutaneous tocilizumab: recent advances for the treatment of rheumatoid arthritis. Expert Opin Drug Deliv.

[19] Burmester GR, Rubbert-Roth A, Cantagrel A (2014). A randomised, double-blind, parallel-group study of the safety and efficacy of subcutaneous tocilizumab versus intravenous tocilizumab in combination with traditional disease-modifying antirheumatic drugs in patients with moderate to severe rheumatoid arthritis (SUMMACTA study). Ann Rheum Dis.

[20] Zink A, Listing J, Kary S (2005). Treatment continuation in patients receiving biological agents or conventional DMARD therapy. Ann Rheum Dis.

[21] Hetland ML, Christensen IJ, Tarp U (2010). Direct comparison of treatment responses, remission rates, and drug adherence in patients with rheumatoid arthritis treated with adalimumab, etanercept, or infliximab: results from eight years of surveillance of clinical practice in the nationwide Danish DANBIO registry. Arthritis Rheum.

[22] van den Bemt BJF, Gettings L, Domańska B (2019). A portfolio of biologic self-injection devices in rheumatology: how patient involvement in device design can improve treatment experience. Drug Deliv.

[23] Jones G, Hall S, Bird P (2018). A retrospective review of the persistence on bDMARDs prescribed for the treatment of rheumatoid arthritis in the Australian population. Int J Rheum Dis.

[24] Arnett FC, Edworthy SM, Bloch DA (1988). The American Rheumatism Association 1987 revised criteria for the classification of rheumatoid arthritis. Arthritis Rheum.

[25] Kay J, Upchurch KS (2012). ACR/EULAR 2010 rheumatoid arthritis classification criteria. Rheumatology.

[26] Anderson J, Caplan L, Yazdany J (2012). Rheumatoid arthritis disease activity measures: American College of Rheumatology recommendations for use in clinical practice. Arthritis Care Res (Hoboken).

[27] Fransen J, van Riel PL (2005). The Disease Activity Score and the EULAR response criteria. Clin Exp Rheumatol.

[28] Bruce B, Fries JF (2003). The Stanford health assessment questionnaire: dimensions and practical applications. Health Qual Life Outcome.

[29] Sokka T (2005). Assessment of pain in rheumatic diseases. Clin Exp Rheumatol.

[30] Anderson JK, Zimmerman L, Caplan L (2011). Measures of rheumatoid arthritis disease activity: Patient (PtGA) and Provider (PrGA) Global Assessment of Disease Activity, Disease Activity Score (DAS) and Disease Activity Score with 28-Joint Counts (DAS28), Simplified Disease Activity Index (SDAI), Clinical Disease Activity Index (CDAI), Patient Activity Score (PAS) and Patient Activity Score-II (PASII), Routine Assessment of Patient Index Data (RAPID), Rheumatoid Arthritis Disease Activity Index (RADAI) and Rheumatoid Arthritis Disease Activity Index-5 (RADAI-5), Chronic Arthritis Systemic Index (CASI), Patient-Based Disease Activity Score With ESR (PDAS1) and Patient-Based Disease Activity Score without ESR (PDAS2), and Mean Overall Index for Rheumatoid Arthritis (MOI-RA). Arthritis Care Res (Hoboken).

[31] Anderson J, Sayles H, Curtis JR (2010). Converting modified health assessment questionnaire (HAQ), multidimensional HAQ, and HAQII scores into original HAQ scores using models developed with a large cohort of rheumatoid arthritis patients. Arthritis Care Res (Hoboken).

[32] Best JH, Vlad SC, Tominna L (2020). Real-world persistence with tocilizumab compared to other subcutaneous biologic disease-modifying antirheumatic drugs among patients with rheumatoid arthritis switching from another biologic. Rheumatol Ther.

[33] Blum MA, Koo D, Doshi JA (2011). Measurement and rates of persistence with and adherence to biologics for rheumatoid arthritis: a systematic review. Clin Ther.

[34] Overton PM, Shalet N, Somers F (2021). Patient preferences for subcutaneous versus intravenous administration of treatment for chronic immune system disorders: a systematic review. Patient Prefer Adherence.

[35] Sobue Y, Suzuki M, Ohashi Y (2022). Validation of grip strength as a measure of frailty in rheumatoid arthritis. Sci Rep.

[36] Salaffi F, Carotti M, Farah S (2021). Handgrip strength features in rheumatoid arthritis patients assessed using an innovative cylindrical-shaped device: relationships with demographic, anthropometric and clinical variables. J Med Syst.

[37] Barton JL (2009). Patient preferences and satisfaction in the treatment of rheumatoid arthritis with biologic therapy. Patient Prefer Adherence.

[38] Williams EL, Edwards CJ (2006). Patient preferences in choosing anti-TNF therapies-R1. Rheumatology (Oxford).

[39] Chilton F, Collett RA (2008). Treatment choices, preferences and decision-making by patients with rheumatoid arthritis. Musculoskeletal Care.

[40] Kishimoto M, Yamairi F, Sato N (2021). Patient preference for treatment mode of biologics in rheumatoid arthritis: a 2020 web-based survey in Japan. Rheumatol Ther.

[41] Ogata A, Tanimura K, Sugimoto T (2014). Phase III study of the efficacy and safety of subcutaneous versus intravenous tocilizumab monotherapy in patients with rheumatoid arthritis. Arthritis Care Res (Hoboken).

[42] Isaacs JD, Salih A, Sheeran T (2019). Efficacy and safety of subcutaneous tocilizumab in rheumatoid arthritis over 1 year: a UK real-world, open-label study. Rheumatol Adv Pract.

[43] Choy E, Caporali R, Xavier R (2018). Subcutaneous tocilizumab in rheumatoid arthritis: findings from the common-framework phase 4 study programme TOZURA conducted in 22 countries. Rheumatology.

[44] Lauper K, Mongin D, Iannone F (2018). Comparative effectiveness of subcutaneous tocilizumab versus intravenous tocilizumab in a pan-European collaboration of registries. RMD open.

[45] Pomponio G, Tontini C, Angeletti A (2017). AB0400 Efficacy and safety of intravenous and subcutaneous tocilizumab in a cohort of patients affected by rheumatoid arthritis in real-life. Ann Rheum Dis.

[46] Genovese MC, Rubbert-Roth A, Smolen JS (2013). Longterm safety and efficacy of tocilizumab in patients with rheumatoid arthritis: a cumulative analysis of up to 4.6 years of exposure. J Rheumatol.

[47] Singh JA, Cameron C, Noorbaloochi S (2015). Risk of serious infection in biological treatment of patients with rheumatoid arthritis: a systematic review and meta-analysis. Lancet.

