# Supplementary material for: Persistence on subcutaneous tocilizumab as monotherapy or in combination with synthetic disease-modifying anti-rheumatic drugs in rheumatoid arthritis patients in Greece (EMBRACE study): a multicenter, post-marketing, non-interventional, observational trial
Source: Clin Rheumatol. 2024 Mar 12;43(5):1469–78. doi: 10.1007/s10067-024-06916-5 (PMC11018651; doi:10.1007/s10067-024-06916-5)
Supplement: Supplementary file 1 — Supplementary file1 (DOCX 210 KB) [file 10067_2024_6916_MOESM1_ESM.docx]

**Table 1. Previous treatments for RA and reason for discontinuation**

| **Previous Treatments**  **(excl. TCZ SC), no (%)** |  | **Ν= 222** |  |
| --- | --- | --- | --- |
| No |  | 57 (25.7) |  |
| Yes |  | 165 (74.3) |  |
| Number of treatments |  |  |  |
| Mean ± SD |  | 2.64±1.52 |  |
| Median [min, max] |  | 2.0 (1.0-8.0) |  |
|  | **Reason for discontinuation – no (%)** | | |
| **Types of previous treatments,**  **n (%)** | Disease progression | AE | Other |
| csDMARD (n= 126) |  |  |  |
| Hydroxychloroquine (n= 30) | 18 (60) | 7 (23.3) | 5 (16.7) |
| Leflunomide (n= 59) | 28 (47.5) | 20 (33.9) | 11 (18.6) |
| Methotrexate (n= 93) | 32 (34.4) | 45 (48.4) | 16 (17.2) |
| Sulfasalazine (n= 2) | 2 (100) | 0 (0) | 0 (0) |
| Other (n= 13) |  |  |  |
| Ciclosporin | 8 (72.7) | 3 (27.3) | 0 (0) |
| Gold preparations | 2 (100) | 0 (0) | 0 (0) |
| anti-TNFα (n= 94) |  |  |  |
| Adalimumab (n= 40) | 27 (67.5) | 4 (10) | 9 (22.5) |
| Certolizumab (n= 17) | 15 (88.2) | 1 (5.9) | 1 (5.9) |
| Etanercept (n= 40) | 31 (77.5) | 2 (5) | 7 (17.5) |
| Golimumab (n= 23) | 18 (78.3) | 1 (4.3) | 4 (17.4) |
| Infliximab (n= 16) | 7 (43.7) | 5 (31.3) | 4 (25) |
| Other (n= 0) | 0 (0) | 0 (0) | 0 (0) |
| Other biologics (n= 47) |  |  |  |
| Abatacept (n= 41) | 37 (90.3) | 1 (2.4) | 3 (7.3) |
| Anakinra (n= 8) | 6 (75) | 1 (12.5) | 1 (12.5) |
| Rituximab (n= 5) | 1 (20) | 1 (20) | 3 (60) |
| Other (n= 1) |  |  |  |
| Secukinumab | 1 (100) | 0 (0) | 0 (0) |
| Surgery | 14 (6.3) | | |
| Other | 33 (14.9) | | |
| Corticosteroids | 32 (97) | | |
| Prednisolone | 30 (93.8) | | |
| Prednisone | 1 (3.1) | | |
| Methylprednisolone | 1 (3.1) | | |
| Calcium | 1 (3) | | |

**Table 2. Clinical outcome assessments during the study according to lines of treatment**

|  | **Baseline visit**  **(Week 0)** | **Observational period**  **(Week 24)** | **Study completion (Week 52)** |
| --- | --- | --- | --- |
| **TCZ SC 1^st^ line (naïve)** | | | |
| **DAS28 score** | **N=109** | **N=98** | **N=104** |
| Mean ± SD | 5. 5±1.3 | 3.1±1.1 | 2.8±1.7 |
| Median [IQR] | 5.7 [1.8-6.3] | 3.0 [2.3-4.0] | 2.4 [1.6-3.7] |
| **Change in DAS28 score**  **from baseline** (Mean, 95% CI, p-value) |  | -2.3, (-2.5, -2.0); **p<0.0001** | -2.6, (-2.9, -2.3); **p<0.0001** |
| **DAS28 score / RA activity – no (%)** |  |  |  |
| DAS28 < 2.6 (remission) | 6 (5.5) | 40 (40.8) | 57 (54.8) |
| 2.6 ≤ DAS28 ≤ 3.2 (low disease activity) | 1 (0.9) | 17 (17.3) | 10 (9.6) |
| 3.2 ≤ DAS28 ≤ 5.1 (Moderate activity) | 28 (25.7) | 35 (35.7) | 24 (23.1) |
| DAS28 > 5.1 (High activity) | 74 (67.9) | 6 (6.1) | 13 (12.5) |
| **EULAR Response– no (%)** | **N=109** | **N=98** | **N=104** |
| Good | 5 (4.6) | 56 (57.1) | 57 (54.8) |
| Moderate | 39 (35.8) | 35 (35.7) | 25 (24) |
| No response | 65 (59.6) | 7 (7.1) | 22 (21.2) |
| **TCZ CS 2^nd^ line** | | | |
| **DAS28 score** | **N=65** | **N=58** | **N=61** |
| Mean ± SD | 5.7±0.9 | 3.1±1.2 | 2.8±1.6 |
| Median [IQR] | 5. 7 [5.3-6.2] | 3.0 [2.3-4.0] | 2.5 [1.8-3.9] |
| **Change in DAS28 score**  **from baseline** (Mean, 95% CI, p-value) |  | -2.6, (-3.0, -2.3); **p<0.0001** | -2.9, (-3.3, -2.4); **p<0.0001** |
| **DAS28 score / RA activity – no (%)** |  |  |  |
| DAS28 < 2.6 (remission) | 1 (1.5) | 23 (39.7) | 35 (57.3) |
| 2.6 ≤ DAS28 ≤ 3.2 (low disease activity) | 0 (0) | 9 (15.5) | 7 (11.5) |
| 3.2 ≤ DAS28 ≤ 5.1 (Moderate activity) | 10 (15.4) | 22 (37.9) | 12 (19.7) |
| DAS28 > 5.1 (High activity) | 54 (83.1) | 4 (6.9) | 7 (11.5) |
| **EULAR Response– no (%)** | **N=65** | **N=58** | **N=61** |
| Good | 4 (6.2) | 32 (55.2) | 40 (65.6) |
| Moderate | 28 (43.1) | 22 (37.9) | 11 (18) |
| No response | 33 (50.8) | 4 (6.9) | 10 (16.4) |
| **Other lines of treatment** |  |  |  |
| **DAS28 score** | **N=48** | **N=43** | **N=43** |
| Mean ± SD | 5.8±0.9 | 3.4±1.1 | 3.2±1.5 |
| Median [IQR] | 5.7 [5.0-6.5] | 3.1 [2.6-4.0] | 2.9 [2.2-4.1] |
| **Change in DAS28 score**  **from baseline** (Mean, 95% CI, p-value) |  | -2.5, (-2.8, -2.2); **p<0.0001** | -2.7, (-3.1, -2.2); **p<0.0001** |
| **DAS28 score / RA activity – no (%)** |  |  |  |
| DAS28 < 2.6 (remission) | 0 (0) | 11 (25.6) | 18 (41.9) |
| 2.6 ≤ DAS28 ≤ 3.2 (low disease activity) | 0 (0) | 12 (27.9) | 6 (14) |
| 3.2 ≤ DAS28 ≤ 5.1 (Moderate activity) | 15 (31.3) | 17 (39.5) | 14 (32.6) |
| DAS28 > 5.1 (High activity) | 33 (68.8) | 3 (7) | 5 (11.6) |
| **EULAR Response– no (%)** | **N=48** | **N=43** | **N=43** |
| Good | 0 (0) | 21 (48.8) | 19 (44.2) |
| Moderate | 20 (41.7) | 21 (48.8) | 21 (48.8) |
| No response | 28 (58.3) | 1 (2.3) | 3 (7) |

**Table 3: Summary characteristics of the recorded adverse events**

| **Seriousness – *no. (%)*** | ***N=41*** |
| --- | --- |
| Death | 2 (4.9) |
| Life-threatening | 0 (0) |
| Initial/Prolonged hospital admission | 2 (4.9) |
| Congenital anomaly/birth defect | 0 (0) |
| Persistent or significant disability | 2 (4.9) |
| Medically significant | 2 (4.9) |
| Non-serious Adverse Events of Special Interest (AESI) | 8 (19.5) |
| Non-serious | 28 (68.3) |
| **Relationship to study drug – *no. (%)*** | ***N=41*** |
| ***All AEs*** |  |
| Yes | 13 (31.7) |
| No | 17 (41.5) |
| Unknown | 13 (31.7) |
| Not provided | 0 (0) |
| ***Serious AEs*** |  |
| Yes | 2 (4.9) |
| No | 4 (9.8) |
| Unknown | 1 (2.4) |
| Not provided | 0 (0) |
| **Outcome – *no. (%)*** | ***N=41*** |
| Fatal | 2 (4.9) |
| Not recovered/Not resolved | 5 (12.2) |
| Recovered/Resolved | 30 (73.2) |
| Recovered/Resolved with sequelae | 1 (2.4) |
| Recovering/Resolving | 7 (17.1) |
| Unknown | 0 (0) |

AE: Adverse event

**Table 4 Comparison of safety-related information between monotherapy and combination therapy groups**

| **Safety-related information** | **All patients (N,%)** | **Monotherapy**  **(N, %)**  **N=74** | | **Combination therapy (N, %)**  **N=148** | | | **Comparison between groups *(p-value)*** |
| --- | --- | --- | --- | --- | --- | --- | --- |
| Any AE | 41 (18.5) | 13 (31.7) | | 28 (68.3) | | | 0.807 |
| Severe AE | 5 (2.3) | 1 (1.4) | | 4 (2.7) | | | 0.548 |
| Treatment-related AE | 14 (6.3) | 3 (9.1) | | 11 (7.4) | | | 0.308 |
| SAE | 7 (3.2) | 3 (9.1) | | 4 (2.7) | | | 0.486 |
| ISR | 5 (2.3) | 4 (12.1) | | 1 (0.7) | | | **0.013** |
| AESI | 8 (3.6) | 2 (6.1) | | 6 (4.1) | | | 0.650 |
| Deaths | 2 (0.9) | 2 (6.1) | | 0 (0) | | | **0.033** |
| Dose modification due to AE (dose decreased and/or frequency reduced) | 4 (1.8) | 0 (0) | | 4 (2.7) | | | - |
| *tocilizumab dose modification* | 0 (0) | 0 (0) | | 0 (0) | | |  |
| *MTX dose modification* | 2* (0.9) | - | | 2 (1.4) | | |  |
| *Other cs DMARDs dose modification* | 2 (0.9) | - | | 2 (1.4) | | |  |
| Dose discontinuations due to an AE | 29 (13.1) | 8 (24.2) | | 21 (14.2) | | | 0.300 |
| *tocilizumab temporary dose discontinuation* | 6 (2.7) | 0 (0) | | 6 (4.1) | | |  |
| *tocilizumab permanent dose discontinuation* | 15 (6.8) | 8 (24.2) | | 7 (4.7) | | |  |
| *MTX dose temporary discontinuation* | 1 (0.5) | - | | 1 (0.7) | | |  |
| *MTX dose permanent discontinuation* | 5 (2.3) | - | | 5 (3.4) | | |  |
| *Other non-biologic DMARDs temporary dose discontinuation* | 1 (0.5) | - | | 1 (0.7) | | |  |
| *Other non-biologic DMARDs permanent dose discontinuation* | 4 (1.8) | - | | 4 (2.7) | | |  |
| Clinical laboratory abnormality |  |  | |  | | |  |
| Neutropenia Grade 1: <LLN – 1500/mm^3^ or <LLN – 1.5 x 109/L | 1 (0.5) | 0 (0) | | | 1 (0.7) |  |  |
| -Neutropenia Grade 2: <1500 – 1000/mm^3^ or <1.5 – 1.0 x 109/L | 0 (0) | 0 (0) | | | 0 (0) |  |  |
| Neutropenia Grade 3: <1000 – 500/mm^3^ or <1.0 – 0.5 x 109/L | 0 (0) | 0 (0) | | | 0 (0) |  |  |
| Neutropenia Grade 4: <500/mm^3^ or <0.5 x 109/L | 0 (0) | 0 (0) | | | 0 (0) |  |  |
| Treatment-emergent ALT or AST > 3 × ULN | 0 (0) | 0 (0) | | | 0 (0) |  |  |
| Treatment-emergent ALT or AST > 3 × ULN in combination with total bilirubin > 2 × the ULN | 0 (0) | 0 (0) | | | 0 (0) |  |  |
| Treatment-emergent ALT or AST > 5 × ULN | 0 (0) | 0 (0) | | | 0 (0) |  |  |
| No change in Total cholesterol (TC)* | 46 (20.7) | 17 (51.5) | | | 29 (19.6) |  |  |
| Change to ≥240 mg/dL^†^ (≥6.21 mmol/L) | 24 (10.8) | | 5 (15.2) | | 19 (12.8) |  |  |
| No change in High-density lipoprotein (HDL-C)^*^ | 38 (17.1) | | 14 (42.4) | | 24 (16.2) |  |  |
| Change to >60 mg/dl (≥1.56 mmol/L) ^*^ | 45 (20.3) | | 11 (33.3) | | 34 (23) |  |  |
| No change in Low-density lipoprotein (LDL-C) ^*^ | 41 (18.5) | | 16 (48.5) | | 25 (16.9) |  |  |
| Change to >160 mg/dl (≥4.12 mmol/L) ^*^ | 16 (7.2) | | 2 (6.1) | | 14 (9.5) |  |  |

*irrespective of AE(s)

AE: Adverse event; AESI: Adverse event of special interest; ISR: Injection site reaction; SAE: Serious adverse event; ULN: Upper level normal

**Table 5.** **Summary of all the recorded Safety Events by System Organ Class (SOC) and Preferred Term (PT) and by causality to tocilizumab**

| **Preferred term by SOC –** **no. (%)** | **Total participants with AE(s) (N=41)** | | **Total events (N=62)** | |
| --- | --- | --- | --- | --- |
|  | **R** | **NR/UNK** | **R** | **NR/UNK** |
| *Blood and lymphatic system disorders (N=4, 5)* |  |  |  |  |
| Leukopenia | 0 (0) | 3 (1.4) | 0 (0) | 3 (4.8) |
| Neutropenia | 1 (0.5) | 0 (0) | 1 (1.6) | 0 (0) |
| Thrombocytopenia | 1 (0.5) | 0 (0) | 1 (1.6) | 0 (0) |
| *Gastrointestinal disorders (N=3, 3)* |  |  |  |  |
| Diarrhea | 1 (0.5) | 1 (0.5) | 1 (1.6) | 1 (1.6) |
| Mouth ulceration | 0 (0) | 1 (0.5) | 0 (0) | 1 (1.6) |
| *General disorders and administration site conditions (N=4, 4)* |  |  |  |  |
| Death | 0 (0) | 1 (0.5) | 0 (0) | 1 (1.6) |
| Drug ineffective | 1 (0.5) | 0 (0) | 1 (1.6) | 0 (0) |
| Injection site rash | 1 (0.5) | 0 (0) | 1 (1.6) | 0 (0) |
| Pyrexia | 0 (0) | 1 (0.5) | 0 (0) | 1 (1.6) |
| *Infections and infestations (N=5, 6)* |  |  |  |  |
| Anal abscess | 0 (0) | 1 (0.5) | 0 (0) | 1 (1.6) |
| Bronchitis | 1 (0.5) | 0 (0) | 1 (1.6) | 0 (0) |
| Infection | 1 (0.5) | 0 (0) | 1 (1.6) | 0 (0) |
| Localised infection | 0 (0) | 1 (0.5) | 0 (0) | 1 (1.6) |
| Onychomycosis | 0 (0) | 1 (0.5) | 0 (0) | 1 (1.6) |
| Urinary tract infection | 0 (0) | 1 (0.5) | 0 (0) | 1 (1.6) |
| *Injury, poisoning and procedural complications (N=1, 1)* |  |  |  |  |
| Limb injury | 0 (0) | 1 (0.5) | 0 (0) | 1 (1.6) |
| *Investigations (N=6, 11)* |  |  |  |  |
| Alanine aminotransferase increased | 2 (0.9) | 0 (0) | 2 (3.2) | 0 (0) |
| Aspartate aminotransferase increased | 2 (0.9) | 0 (0) | 2 (3.2) | 0 (0) |
| Blood alkaline phosphatase increased | 1 (0.5) | 0 (0) | 1 (1.6) | 0 (0) |
| Blood cholesterol increased | 1 (0.5) | 0 (0) | 1 (1.6) | 0 (0) |
| Blood creatine increased | 1 (0.5) | 0 (0) | 1 (1.6) | 0 (0) |
| Blood glucose increased | 0 (0) | 1 (0.5) | 0 (0) | 1 (1.6) |
| Blood uric acid decreased | 1 (0.5) | 0 (0) | 1 (1.6) | 0 (0) |
| Liver function test increased | 0 (0) | 1 (0.5) | 0 (0) | 1 (1.6) |
| Neutrophil count decreased | 1 (0.5) | 0 (0) | 1 (1.6) | 0 (0) |
| Platelet count decreased | 0 (0) | 1 (0.5) | 0 (0) | 1 (1.6) |
| White blood cell count decreased | 1 (0.5) | 1 (0.5) | 1 (1.6) | 1 (1.6) |
| *Metabolism and nutrition disorders (N=8, 9)* |  |  |  |  |
| Dyslipidemia | 0 (0) | 5 (2.3) | 0 (0) | 5 (8.1) |
| Gout | 0 (0) | 1 (0.5) | 0 (0) | 1 (1.6) |
| Hyperlipidemia | 1 (0.5) | 0 (0) | 1 (1.6) | 0 (0) |
| Hyponatremia | 0 (0) | 1 (0.5) | 0 (0) | 1 (1.6) |
| Metabolic syndrome | 0 (0) | 1 (0.5) | 0 (0) | 1 (1.6) |
| *Musculoskeletal and connective tissue disorders (N=4, 4)* |  |  |  |  |
| Arthralgia | 1 (0.5) | 0 (0) | 1 (1.6) | 0 (0) |
| Fibromyalgia | 0 (0) | 2 (0.9) | 0 (0) | 2 (3.2) |
| Intervertebral disc protrusion | 0 (0) | 1 (0.5) | 0 (0) | 1 (1.6) |
| *Nervous system disorders (N=1, 1)* |  |  |  |  |
| Myelopathy | 0 (0) | 1 (0.5) | 0 (0) | 1 (1.6) |
| *Psychiatric disorders (N=1, 1)* |  |  |  |  |
| Insomnia | 0 (0) | 1 (0.5) | 0 (0) | 1 (1.6) |
| *Respiratory, thoracic and mediastinal disorders (N=2, 2)* |  |  |  |  |
| Pulmonary edema | 0 (0) | 1 (0.5) | 0 (0) | 1 (1.6) |
| Pulmonary sarcoidosis | 0 (0) | 1 (0.5) | 0 (0) | 1 (1.6) |
| *Skin and subcutaneous tissue disorders (N=9, 9)* |  |  |  |  |
| Alopecia | 0 (0) | 2 (0.9) | 0 (0) | 2 (3.2) |
| Dermatitis | 0 (0) | 1 (0.5) | 0 (0) | 1 (1.6) |
| Dermatitis allergic | 1 (0.5) | 0 (0) | 1 (1.6) | 0 (0) |
| Eczema | 0 (0) | 1 (0.5) | 0 (0) | 1 (1.6) |
| Rash | 2 (0.9) | 2 (0.9) | 2 (3.2) | 2 (3.2) |
| *Surgical and medical procedures (N=1, 1)* |  |  |  |  |
| Neurosurgery | 0 (0) | 1 (0.5) | 0 (0) | 1 (1.6) |
| *Vascular disorders (N=2, 2)* |  |  |  |  |
| Aortic stenosis | 0 (0) | 1 (0.5) | 0 (0) | 1 (1.6) |
| Hypertension | 0 (0) | 1 (0.5) | 0 (0) | 1 (1.6) |

R: related, NR: Not-related, Unk: Unknown

**Table 6** **Abnormal laboratory tests recorded at each visit**

| **Laboratory tests** | **Baseline Visit**  **N=222** | | **Observational period visit (Week 24)**  **N=216** | | **Study completion visit (Week 52)**  **N=208** | |
| --- | --- | --- | --- | --- | --- | --- |
|  | Clinically significant **– *no (%)*** | | Clinically significant **– *no (%)*** | | Clinically significant **– *no (%)*** | |
|  | Yes | No | Yes | No | Yes | No |
| **White blood cells** | 0 (0) | 190 (85.6) | 1 (0.5) | 172 | 0 (0) | 159 (76.4) |
| **Neutrophils** | 0 (0) | 165 (74.3) | 0 (0) | 152 (70.4) | 0 (0) | 145 (69.7) |
| **Eosinophils** | 0 (0) | 149 (67.1) | 0 (0) | 138 (63.9) | 0 (0) | 126 (60.6) |
| **Basophils** | 0 (0) | 120 (54.1) | 0 (0) | 110 (50.9) | 0 (0) | 152 (73.1) |
| **Lymphocytes** | 0 (0) | 155 (69.8) | 0 (0) | 142 (65.7) | 0 (0) | 139 (66.8) |
| **Monocytes** | 0 (0) | 145 (65.3) | 0 (0) | 128 (59.3) | 0 (0) | 128 (61.5) |
| **Hemoglobin (Hb)** | 1 (0.5) | 186 (83.8) | 0 (0) | 168 (77.8) | 0 (0) | 154 (74) |
| **Hematocrit (Hct)** | 1 (0.5) | 192 (86.5) | 0 (0) | 177 (81.9) | 0 (0) | 169 (81.3) |
| **Platelets** | 1 (0.5) | 190 (85.6) | 0 (0) | 169 (78.2) | 0 (0) | 160 (76.9) |
| **Potassium** | 0 (0) | 57 (25.7) | 0 (0) | 32 (14.8) | 0 (0) | 34 (16.3) |
| **Sodium** | 0 (0) | 56 (25.2) | 0 (0) | 34 (15.7) | 0 (0) | 32 (15.4) |
| **Calcium** | 0 (0) | 74 (33.3) | 0 (0) | 48 (22.2) | 0 (0) | 52 (25) |
| **Glucose** | 0 (0) | 172 (77.5) | 0 (0) | 145 (67.1) | 0 (0) | 147 (70.7) |
| **Urea** | 0 (0) | 169 (76.1) | 0 (0) | 140 (64.8) | 0 (0) | 131 (63) |
| **Creatinine** | 0 (0) | 186 (83.8) | 1 (0.5) | 154 (71.3) | 0 (0) | 144 (69.2) |
| **AST (SGOT)** | 0 (0) | 191 (86) | 1 (0.5) | 164 (75.9) | 2 (1) | 157 (75.5) |
| **ALT (SGPT)** | 0 (0) | 189 (85.1) | 1 (0.5) | 164 (75.9) | 2 (1) | 156 (75) |
| **ALP** | 0 (0) | 108 (48.6) | 1 (0.5) | 87 (40.3) | 1 (0.5) | 92 (44.2) |
| **γ-GT** | 0 (0) | 129 (58.1) | 1 (0.5) | 97 (44.9) | 1 (0.5) | 102 (49) |
| **Lactate dehydrogenase (LD)** | 0 (0) | 43 (19.4) | 0 (0) | 28 (13) | 0 (0) | 32 (15.4) |
| **Amylase** | 0 (0) | 13 (5.9) | 0 (0) | 7 (3.2) | 0 (0) | 7 (3.4) |
| **Bilirubin Total** | 0 (0) | 23 (10.4) | 0 (0) | 16 (7.4) | 0 (0) | 14 (6.7) |
| **Proteins Total** | 0 (0) | 42 (18.9) | 0 (0) | 24 (11.1) | 0 (0) | 32 (15.4) |
| **Albumin** | 0 (0) | 41 (18.5) | 0 (0) | 22 (10.2) | 0 (0) | 27 (13) |
| **C-reactive protein (CRP)** | 1 (0.5) | 172 (77.5) | 0 (0) | 166 (76.9) | 0 (0) | 160 (76.9) |
| **Erythrocyte sedimentation rate (ESR)** | 1 (0.5) | 182 (82) | 0 (0) | 169 (78.2) | 0 (0) | 172 (82.7) |
| **Folic acid** | 0 (0) | 18 (8.1) | 0 (0) | 21 (9.7) | 0 (0) | 18 (8.7) |
| **Vitamin B_12_** | 0 (0) | 21 (9.5) | 0 (0) | 22 (10.2) | 0 (0) | 23 (11.1) |
| **Iron** | 0 (0) | 30 (13.5) | 0 (0) | 20 (9.3) | 0 (0) | 22 (10.6) |
| **Ferritin** | 0 (0) | 46 (20.7) | 0 (0) | 34 (15.7) | 0 (0) | 33 (15.9) |
| **Total Cholesterol** | 0 (0) | 142 (64) | 1 (0.5) | 134 (62) | 1 (0.5) | 121 (58.2) |
| **Triglycerides (TG)** | 0 (0) | 124 (55.9) | 1 (0.5) | 110 (50.9) | 1 (0.5) | 103 (49.5) |
| **Low-density cholesterol (LDL-C)** | 0 (0) | 90 (40.5) | 1 (0.5) | 82 (38) | 1 (0.5) | 83 (39.9) |
| **High density cholesterol (HDL-C)** | 0 (0) | 100 (45) | 0 (0) | 99 (45.8) | 0 (0) | 89 (42.8) |
| **Rheumatoid factor (RF)** | 1 (0.5) | 128 (57.7) | 0 (0) | 33 (15.3) | 0 (0) | 31 (14.9) |
| **anti-CCP** | 1 (0.5) | 96 (43.2) | 0 (0) | 20 (9.3) | 0 (0) | 17 (8.2) |

*ALP: Alkaline phosphatase; ALT: Alanine aminotransferase; AST: aspartate aminotransferase; CCP: Citric citrullinated peptide; GT: glutamy transferase*

**Table 7 Summary of Adverse Events of Special Interest per treatment group and by System Organ Class (SOC) and Preferred Term (PT)**

| **Preferred term by SOC –** **no. (%)** | **Monotherapy** | | **Combination therapy** | |
| --- | --- | --- | --- | --- |
|  | **No. of patients** | **No. of events** | **No. of patients** | **No. of events** |
| *Blood and lymphatic system disorders* |  |  | 2 | 3 |
| Leukopenia |  |  | 1 | 1 |
| Neutropenia |  |  | 1 | 1 |
| Thrombocytopenia |  |  | 1 | 1 |
| *General disorders and administration site conditions* | 1 | 1 |  |  |
| Pyrexia | 1 | 1 |  |  |
| Infections and infestations |  |  | 2 | 3 |
| Bronchitis |  |  | 1 | 1 |
| Infection |  |  | 1 | 1 |
| Urinary tract infection |  |  | 1 | 1 |
| *Investigations* |  |  | 3 | 9 |
| Alanine aminotransferase increased |  |  | 2 | 2 |
| Aspartate aminotransferase increased |  |  | 2 | 2 |
| Blood alkaline phosphatase increased |  |  | 1 | 1 |
| Blood cholesterol increased |  |  | 1 | 1 |
| Blood creatine increased |  |  | 1 | 1 |
| Blood uric acid decreased |  |  | 1 | 1 |
| Platelet count decreased |  |  | 1 | 1 |
| *Musculoskeletal and connective tissue disorders* | 1 | 1 |  |  |
| Fibromyalgia | 1 | 1 |  |  |
| **Total (N,%)^†^** | 2 (2.7) | 2 (11.8) | 6 (4.1) | 15 (33.3) |

**Each participant could experience more than one adverse event;*

***^†^****N of patients and events within each category. % is of the total number of monotherapy (N=74)/combination therapy (N=148) patients and of the total number of events per treatment group (N=17 and N=45 respectively)*

**Table 8 Summary of Serious Adverse Events per treatment group and by System Organ Class (SOC) and Preferred Term (PT)**

| **Preferred term by SOC – no. (%)** | **Monotherapy** | | **Combination therapy** | |
| --- | --- | --- | --- | --- |
|  | **No. of patients*** | **No. of events** | **No. of patients** | **No. of events** |
| *General disorders and administration site conditions* |  |  |  |  |
| Death | 1 | 1 |  |  |
| Injection site rash | 1 | 1 |  |  |
| *Investigations* |  |  |  |  |
| Neutrophil count decreased |  |  | 1 | 1 |
| White blood cell count decreased |  |  | 1 | 1 |
| *Metabolism and nutrition disorders* |  |  |  |  |
| Metabolic syndrome |  |  | 1 | 1 |
| *Musculoskeletal and connective tissue disorders* |  |  |  |  |
| Intervertebral disc protrusion |  |  | 1 | 1 |
| *Nervous system disorders* |  |  |  |  |
| Myelopathy |  |  | 1 | 1 |
| *Respiratory, thoracic and mediastinal disorders* |  |  |  |  |
| Pulmonary oedema | 1 | 1 |  |  |
| *Surgical and medical procedures* |  |  |  |  |
| Neurosurgery |  |  | 1 | 1 |
| *Vascular disorders* |  |  |  |  |
| Aortic stenosis |  |  | 1 | 1 |
| Total (N, %)^†^ | 3 (4.1) | 3 (17.6) | 4 (2.7) | 7 (15.6) |

*^*^Each participant could experience more than one adverse event.*

*N represents number of patients and events within each category.*

*% represents the total number of patients receiving monotherapy (N=74)/combination therapy (N=148), and the total number of events per treatment group (N=17 and N=45, respectively)*

**Table 9 Summary characteristics of the recorded safety events by persistent-non-persistent subpopulations**

| **AE characterization** | **Persistent population – no. (%)**  **N=27** | **Non-persistent population – no. (%)**  **N=14** | | |  |
| --- | --- | --- | --- | --- | --- |
| **Seriousness – *no. (%)*** |  |  | | | |
| Death | 0 (0) | 2 (14.3) | | | |
| Life-threatening | 0 (0) | 0 (0) | | | |
| Initial/Prolonged hospital admission | 1 (3.7) | 1 (7.1) | | | |
| Congenital anomaly/birth defect | 0 (0) | 0 (0) | | | |
| Persistent or significant disability | 1 (3.7) | 1 (7.1) | | | |
| Medically significant | 2 (7.4) | 0 (0) | | | |
| Non-serious Adverse Events of Special Interest (AESI) | 5 (18.5) | 3 (21.4) | | | |
| Non serious | 20 (74.1) | 8 (57.1) | | | |
| **Relationship to study drug – *no. (%)*** |  |  | | | |
| Yes | 7 (25.9) | 7 (50) | | | |
| No | 13 (48.2) | 5 (35.7) | | | |
| Unknown | 11 (40.7) | 2 (14.3) | | | |
| Not provided | 0 (0) | 0 (0) | |  |  |
| **Outcome – *no. (%)*** |  |  |  |  |  |
| Fatal | 0 (0) | 2 (14.3) | | | |
| Not recovered/Not resolved | 3 (11.1) | 2 (14.3) | | | |
| Recovered/Resolved | 21 (77.8) | 9 (64.3) | | | |
| Recovered/Resolved with sequelae | 1 (3.7) | 0 (0) | | | |
| Recovering/Resolving | 5 (18.5) | 2 (14.3) | | | |
| Unknown | 0 (0) | 0 (0) | | | |

**Table 10 Overview of AEs for persistent and non-persistent population**

| **Safety-related information** | **Persistent population (N, %)**  **N=189** | **Non-persistent population (N, %)**  **N=33** | **Comparison between groups *(p-value)*** |
| --- | --- | --- | --- |
| Any AE | 27 (14.3) | 14 (42.4) | <0.0001 |
| Severe AE | 3 (1.6) | 2 (6.1) | 0.161 |
| Treatment-related AE | 7 (3.7) | 7 (21.2) | 0.001 |
| SAE | 3 (1.6) | 4 (12.1) | 0.010 |
| ISR | 0 (0) | 5 (15.2) | <0.0001 |
| AESI | 5 (2.6) | 3 (9.1) | 0.099 |
| Deaths | 0 (0) | 2 (6.1) | 0.022 |
| Dose modification due to AE (dose decreased and/or frequency reduced) | 3 (1.6) | 1 (3) | 0.477 |
| *tocilizumab dose modification* | 0 (0) | 0 (0) |  |
| *MTX dose modification* | 1 (0.5) | 1 (3) |  |
| *Other non-biologic DMARDs dose modification* | 2 (1.1) | 0 (0) |  |
| Dose discontinuations due to an AE | 16 (8.5) | 13 (39.4) | <0.0001 |
| *tocilizumab temporary dose discontinuation* | 6 (3.2) | 0 (0) |  |
| *tocilizumab permanent dose discontinuation* | 3 (1.6) | 12 (36.4) |  |
| *MTX dose temporary discontinuation* | 1 (0.5) | 0 (0) |  |
| *MTX dose permanent discontinuation* | 4 (2.1) | 1 (3) |  |
| *Other non-biologic DMARDs temporary dose discontinuation* | 1 (0.5) | 0 (0) |  |
| *Other non-biologic DMARDs permanent dose discontinuation* | 4 (2.1) | 0 (0) |  |
| Clinical laboratory abnormality | 1 (0.5) | 0 (0) | - |
| Neutropenia Grade 1: <LLN – 1500/mm^3^ or <LLN – 1.5 x 109/L | 1 (0.5) | 0 (0) |  |
| Neutropenia Grade 2: <1500 – 1000/mm^3^ or <1.5 – 1.0 x 109/L | 0 (0) | 0 (0) |  |
| Neutropenia Grade 3: <1000 – 500/mm3 or <1.0 – 0.5 x 109/L | 0 (0) | 0 (0) |  |
| Neutropenia Grade 4: <500/mm^3^ or <0.5 x 109/L | 0 (0) | 0 (0) |  |
| Treatment-emergent ALT or AST > 3 × ULN | 0 (0) | 0 (0) |  |
| Treatment-emergent ALT or AST > 3 × ULN in combination with total bilirubin > 2 × the ULN | 0 (0) | 0 (0) |  |
| Treatment-emergent ALT or AST > 5 × ULN | 0 (0) | 0 (0) |  |
| No change in Total cholesterol* (TC) | 30 (15.9) | 16 (48.5) |  |
| Change to ≥240 mg/dL* (≥6.21 mmol/L) | 23 (12.2) | 1 (3) |  |
| No change in High-density lipoprotein* (HDL-C) | 27 (14.3) | 11 (33.3) |  |
| Change to >60 mg/dl* (≥1.56 mmol/L) | 44 (23.3) | 1 (3) |  |
| No change in Low-density lipoprotein* (LDL-C) | 10 (5.3) | 31 (93.9) |  |
| Change to >160 mg/dl* (≥4.12 mmol/L) | 15 (7.9) | 1 (3) |  |

**Irrespective of AEs; AE: Adverse event; AESI: Adverse event of special interest; ISR: Injection site reaction; SAE: Serious adverse event; ULN: Upper level normal*

**Table 11 Annualized Rate of AEs, SAEs and AESI**

|  | |  | N=222 (%) |
| --- | --- | --- | --- |
| Adverse Events | Total patients with ≥1 AE n (%) | | 41 (18.5) |
|  | Rate of AEs (per 100 PY) | | 1.51 |
| Serious Adverse Events | Total patients with ≥1 SAE n (%) | | 7 (3.2) |
|  | Rate of SAEs (per 100 PY) | | 1.43 |
| Adverse Events of Special Interest | Total patients with ≥1 AESI n (%) | | 8 (3.6) |
|  | Rate of AESI (per 100 PY) | | 2.13 |
| Infections | Total patients with ≥1 AE n (%) | | 5 (2.3) |
|  | Rate of AEs (per 100 PY) | | 1.20 |
| Serious infections | Total patients with ≥1 SI n (%) | | 0 (0) |
|  | Rate of SIs (per 100 PY) | | - |

AE: Adverse event; AESI: Adverse event of special interest; PY: patient years

**Table 12 Subcutaneous tocilizumab dose modifications**

| **Dose modifications in tocilizumab –** **no. (%)** | **N=222** |
| --- | --- |
| No modification | 222 (100) |
| ≤ 2 modifications | 0 (0) |
| > 2 modifications | 0 (0) |
| **Discontinuation of tocilizumab –** **no. (%)** | **38 (17.1)** |
| At least 1 temporary discontinuation | 7 (3.2) |
| **tocilizumab received at least for 40 weeks –** **no. (%)** | 194 (87.4) |
| **Reason for modification/discontinuation** **–** **no. (%)** | **N=222** |
| Disease progression | 11 (5.0) |
| Adverse event | 18 (8.1) |
| Alopecia | 2 (11.1) |
| ALT increased, AST increased | 1 (5.5) |
| Dermititis | 1 (5.5) |
| Dermitits Allergic | 1 (5.5) |
| Diarrhea | 2 (11.1) |
| Gout | 1 (5.5) |
| Injection site rash | 1 (5.5) |
| Leukopenia | 3 (16.6) |
| Limb inury | 1 (5.5) |
| Liver function test increased | 1 (5.5) |
| Metabolic syndrome | 1 (5.5) |
| Pyrexia | 1 (5.5) |
| Rash | 2 (11.1) |
| Other | 12 (5.4) |


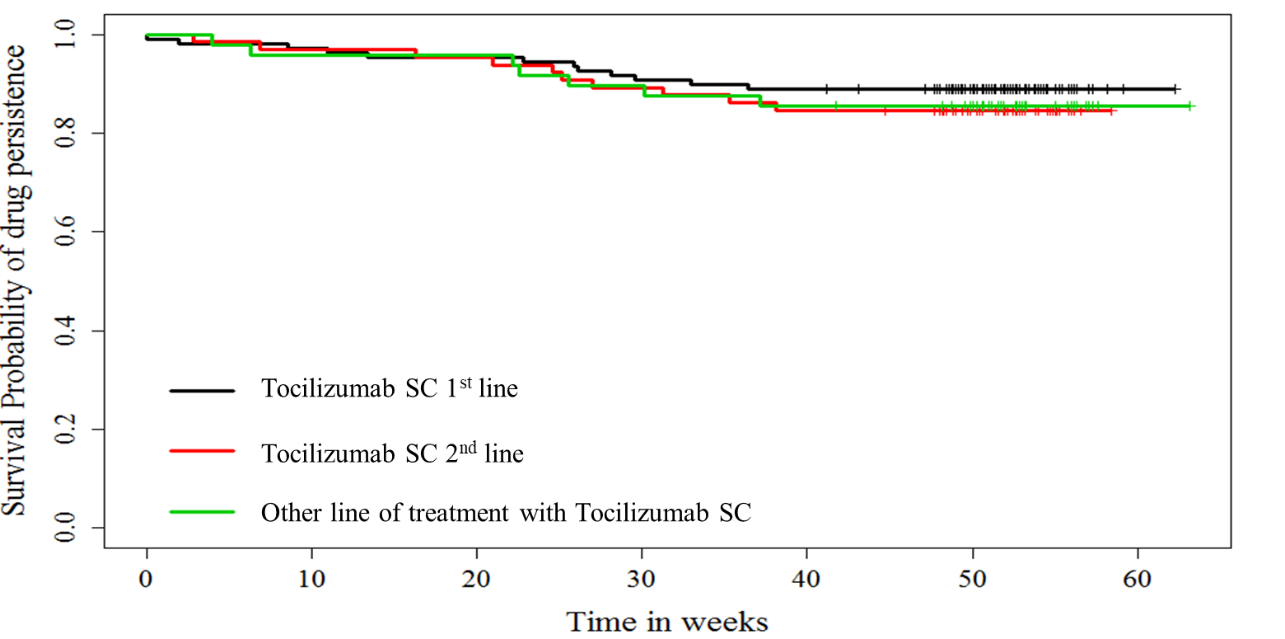


**Fig 1** Kaplan-Meier estimates of TCZ SC persistence per line of treatment. Censored observations are marked with “+”.


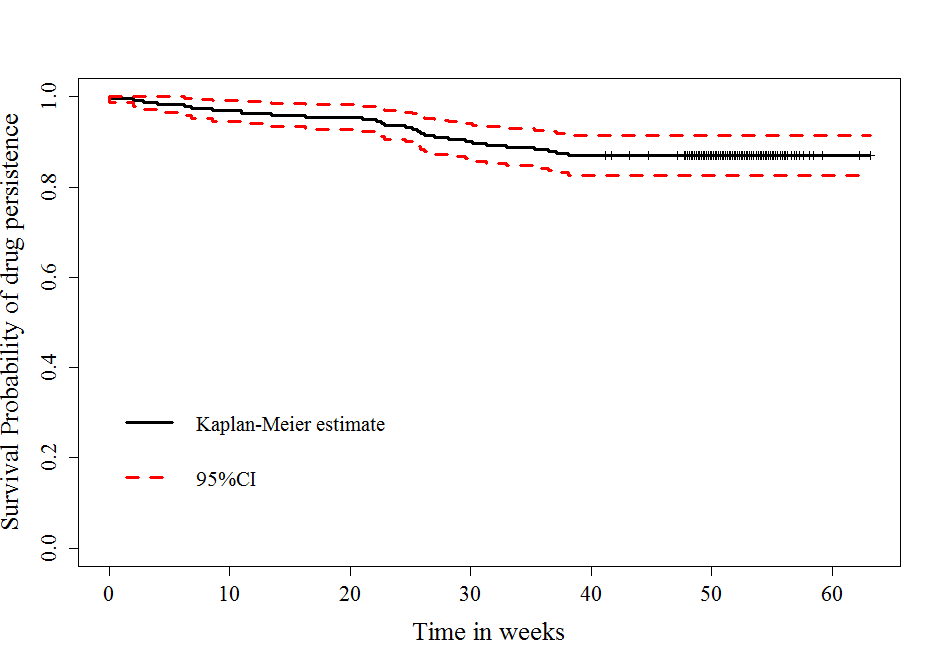


**Fig 2** Overall drug survival – Kaplan-Meier estimates and 95% CIs. Censored observations are marked with “+”.
